# Seroconversion Rate After SARS-CoV-2 Infection and Two Doses of Either ChAdOx1-nCOV COVISHIELD™ or BBV-152 COVAXIN™ Vaccination in Renal Allograft Recipients: An Experience of Two Public and Private Tertiary Care Center

**DOI:** 10.3389/fimmu.2022.911738

**Published:** 2022-06-30

**Authors:** Narayan Prasad, Shyam Bihari Bansal, Brijesh Yadav, Neha Manhas, Deependra Yadav, Sonam Gautam, Ravishankar Kushwaha, Ankita Singh, Dharmendra Bhadauria, Monika Yachha, Manas Ranjan Behera, Anupama Kaul

**Affiliations:** ^1^ Department of Nephrology and Renal Transplantation, Sanjay Gandhi Postgraduate Institute of Medical Sciences, Lucknow, India; ^2^ Medanta The Medicity Hospital, Gurgaon, India

**Keywords:** vaccination, anti- SARS-CoV-2 antibody, humoral immunity, COVISHIELD™, COVAXIN™

## Abstract

**Introduction:**

Vaccination is an effective strategy for preventing SARS-CoV-2 infection and associated mortality. Renal Transplant Recipients (RTRs) are vulnerable to acquiring infection and high mortality due to their immunocompromised state. Varying responses to the different vaccines, depending on types of vaccines and population, have been reported. Vaccines supply is also limited. The current study evaluated the seroconversion rate after SARS-CoV-2 infection and 2 doses of either COVAXIN™ or COVISHIELD™ vaccination in RTR.

**Methods:**

The serum anti-SARS-CoV-2 spike protein neutralizing antibody titer was measured in 370 RTRs who acquired SARS-CoV-2 infection (n=172), yet not vaccinated; and those vaccinated with COVAXIN™ (n=78), and COVISHIELD™ (n=120) by chemiluminescence microparticle immunoassay methods from serum.

**Result:**

Overall, the seroconversion rate either after vaccination or infection was 85.13% (315/370). The vaccine-associated seroconversion was 80.30% (159/198). SARS-CoV-2 infection-associated seroconversion was 90.69% (156/172), COVISHIELD™ associated seroconversion was 79.2% (95/120), and COVAXIN™ associated seroconversion was 82.05% (64/78). The median IgG titer in the SARS-CoV-2 infection group was 646.50 AU/ml (IQR: 232.52-1717.42), in the COVAXIN™ group was 1449.75 AU/ml (IQR: 400.0-3068.55), and the COVISHIELD™ vaccination group was 1500.51 AU/ml (IQR: 379.47-4938.50). The seroconversion rate and antibody titers were similar irrespective of the place of sampling. Patient’s age-associated seroconversion in <45 years was 88.01% (213/242), 45.1-60 years was 83.18% (94/113), and > 60 years was 58.3% (7/12).

**Conclusions:**

Both infection and vaccination induce robust antibody formation in RTRs. The seroconversion rate after SARS-CoV-2 infection was higher but with a lower antibody titer than vaccines. The vaccines, COVAXIN™ and COVISHIELD™, induce more elevated antibody titers than natural infection. The seroconversion rate and antibody titer in Indian RTRs appears to be better than in the western population, irrespective of their vaccination status.

## Introduction

Vaccination is one of the most effective strategies in preventing SARS-CoV-2 infection and transmission during a pandemic (1–3). There has been the emergence of multiple SARS-CoV-2 variants and repeated infection episodes in several people. However, the vaccines prevented morbidity, hospitalization, and mortality of patients suffering from coronavirus diseases 19 (COVID19). Several vaccines have been developed against the SARS-CoV-2 virus in multiple countries, including India. The high demand for vaccines from across the world has limited the availability of vaccines in low resources countries (4). Well-validated mRNA-based vaccines BNT162b2 (Pfizer-BioNTech, USA) are mainly limited to developed countries. The vaccines have shown high seroconversion rate in the general population up to the tune of 95%, however, had a poor seroconversion rate in renal transplant recipient (RTR) (5–7). Data of mRNA-based vaccination showed a 48% of seroconversion rate in RTRs after the 28^th^ day of the 2^nd^ dose of vaccination (1, 6, 8).

Adenovirus vector-based vaccines ChAdOx1-nCOV (COVISHIELD™, AstraZeneca–Oxford University and Serum Institute, India) and inactivated whole virus-based BBV-152 (COVAXIN™, The Bharat Biotech, India) vaccine are available in India. These vaccines have also shown a good seroconversion rate in a healthy population (2). However, the seroconversion data is limited to a small single-center study in RTRs (9). A single-center study showed seroconversion of about 70% in RTRs, which is higher than that reported from mRNA-based vaccines (1, 10). A lesser amount of antibody formation and poor seroconversion rate after vaccination and SARS-CoV-2 infection is expected in RTRs because of immunosuppressive medicines (1, 6, 7). A reduction of immunosuppression may boost the antibody formation in these patients, although this may pose patients at risk of allograft rejection.

Few studies have reported the incidence of allograft rejection after the vaccination (1, 11, 12). Notably, a 100% seroconversion rate was observed after a single vaccination dose in RTRs, infected previously with SARS-CoV-2 (13, 14). Elicitation of antibodies after vaccination depends on the (i) nature of the antigens and adjuvants, (ii) dose of antigen, and (iii) mode of vaccine delivery (15). The antigenic material used in mRNA-based, vector-based, and inactivated whole virus-based vaccines are known to be different. Therefore, it may be interesting to hypothesize and study whether a whole inactivated virus-based vaccine-like BBV-152 (COVAXIN™) may be more effective in immunocompromised RTRs, who are at a higher risk of acquiring SARS-CoV-2 infection and develop severe COVID-19 and related mortality. The cause for such heterogeneous response to vaccination in RTRs may vary on the duration and degree of immunosuppression (8). Developing and testing the efficacy of other vaccines in antibody formation remained a high priority research area. In the present two center studies, we aimed to study the overall seroconversion rate after (i) two doses of anti-SARS-CoV-2 vaccination and (ii) SARS-CoV-2 infection among non-vaccinated RTRs. Further, we have carefully evaluated the potential association of clinical variables influencing antibody formation in RTRs.

## Materials and Methods

### Patient Population

A total of 370 RTRs were included in the study from two centers, Medanta Medicity hospital Gurugram, New Delhi, India, a private sector tertiary care center, and Sanjay Gandhi Postgraduate Institute of Medical Sciences, Lucknow, India, a public sector tertiary care teaching institute between 1^st^ June 2021 to 30^th^ November 2021. This study was approved by the Institutional Ethics Committee and adhered to the ethical standards of the declaration of Istanbul and Helsinki. The ethics approval code was 2021-36-IP-EXP-36. All patients were reverse transcriptase-polymerase chain reaction (RT-PCR) negative at the time of sample collection. The demographic and clinical details were noted at the time of sample collection from the patient’s medical record. The prior history of SARS-CoV-2 infection was 78 (range, 56-90) days. Vaccination history and associated side effects fever, myalgias, headache, back pain, body ache, and giddiness were obtained from each participating individual. The type and dose of vaccines were confirmed from the vaccination certificate issued by the Ministry of Health and Family Welfare, Government of India. RTRs who had SARS-CoV-2 infection and yet not received vaccines and those who received two doses of vaccines were asked for the blood sampling for anti-SARS-CoV-2 spike protein IgG measurement. The mean gap between two doses of either brand of vaccination and samples collection was 21.10 ± 4.27 days. The median interval between 1^st^ and 2^nd^ dose for COVISHIELD™ vaccine was 69 (range, 42-112) days, and for COVAXIN™, it was 36 (range, 28-42) days.

For the analysis purpose, patients were categorized into three groups. Group-1, those who had a history of SARS-CoV-2 infection yet did not receive any dose of vaccines (n=172). Group-2, those who had received 2 doses of COVAXIN™ (n=78), and Group-3, those who had received 2 doses of COVISHIELD™ vaccine (n=120).

### Anti-SARS-CoV-2 Spike Protein IgG Titer Measurement by Chemiluminescence Immunoassay Methods

A five ml blood sample was collected in a plain vial with blood clot activating factors and centrifugation at 1500RPM for 5 minutes. The serum was separated and stored at -80^0^C. Anti-SARS-CoV-2 spike protein IgG titer was determined using the chemiluminescent magnetic microparticle Immunoassay (CMIA) analyzer per the manufacturer’s instruction (Abbott diagnostic, Ireland).

In brief, in this process, the first serum anti-SARS-CoV-2 IgG antibody was captured on an antigen-coated paramagnetic microparticle bead and buffer. The non-specific binding was removed by the washing. The antigen-antibody complex mixture was further incubated with acridinium labeled anti-human IgG conjugate. The complex mixture was again washed with buffer to remove non-specific binding. Further, a pre-trigger and trigger solution of hydrogen peroxide and sodium hydroxide was added, resulting in a chemiluminescent mixture on the Architect platform (Abbott diagnostic, Ireland). The intensity of the chemiluminescent mixture was measured in a relative light unit (RLU) that was directly proportional to the concentration of anti-SARS-CoV-2 antibody present in the serum. The sample’s RLU values were normalized with the calibrator RLU as per the World Health Organization standard (16, 17).

### Statistical Analysis

Statistical analysis was performed using the SPSS software version 20 (IBM corporation, Armonk, NY, USA). Kruskal Wallis test was used to compare the median of nonparametrically distributed variables between the groups. Median and interquartile range was calculated for the antibody titer. For the comparison of continuous variables among the group, a one-way analysis of variance (ANOVA) was applied. The mean and standard deviation was calculated. The Chi-square test or the Fischer exact test was used per the application required to compare the categorical variables. Multivariate analysis was also performed for variables predicting seroconversion. Graphs were plotted with Prism version 8 for Windows, GraphPad Software, La Jolla, CA, USA.

## Results

### Demographic and Clinical Characteristics of the Patients

Demographic and clinical profiles of the patients are given in **Table 1**. Eighty-five (317/370) percent of patients were male, and 46.48% (172/370) had previous SARS-CoV-2 infection without a history of any dose of vaccination. Of these, 21.08% (78/370) of patients were vaccinated with COVAXIN™, and 32.43% (120/370) patients were vaccinated with COVISHIELD™. The mean age of the patients was 40.84 years and the median post-transplant period to sample collection for testing anti-SARS-CoV-2 spike protein IgG was 78.99 months. All patients were live-related renal allograft recipients, and the majority, 93.78% (347/370) were ABO compatible RTRs.

**Table 1:** Demographic and clinical characteristic of patients in SARS-CoV-2 infection and Vaccination.

Characteristics		Total	SARS-CoV-2 infection(n=172)	COVAXIN™Vaccination(n=78)	COVISHIELD™Vaccination(n=120)	P Value
Age (Years)		40.84 ± 10.84	39.65 ± 10.02	41.87 ± 10.79	41.89 ± 11.86	0.14*
Male/Female		317/53	150/22	65/13	102	0.69**
ABOc/ABOi		347/23	164/8	76/2	107/13	0.032**
Post-transplant interval in Month (mean ± SD)		78.99 ± 52.3	82.19 ± 56.18	85.61 ± 54.35	70.09 ± 43.61	0.068*
BMI(Kg/M^2^)		23.72 ± 4.82	23.73 ± 5.16	23.41 ± 3.14	23.90 ± 5.22	0.78*
Hemoglobin(g/dl)		12.54 ± 2.05	13.02 ± 1.85	11.39 ± 2.38	12.61 ± 1.80	<0.001*
BUN (mg/dl)		23.76 ± 11.81	19.83 ± 7.82	27.09 ± 13.38	27.22 ± 13.67	0.001*
Baseline serum creatinine(mg/dl)		0.87 ± 0.41	1.03 ± 0.40	0.71 ± 0.39	0.76 ± 0.35	<0.001*
Serum creatinine(mg/dl)		1.34 ± 0.60	1.47 ± 0.79	1.23 ± 0.29	1.23 ± 0.34	0.001*
TLC (X10^3/^µl)		7.63 ± 2.46	8.26 ± 2.58	6.75 ± 2.06	7.30 ± 2.30	<0.001*
eGFR (ml/min)		73.41 ± 34.62	70.42 ± 45.17	75.92 ± 21.21	76.05 ± 21.68	0.30*
Tacrolimus level(µg/l)		5.44 ± 1.94	5.56 ± 2.18	5.19 ± 1.09	5.45 ± 2.00	0.37*
Systolic BP (mmHg)		133.17 ± 14.37	130.13 ± 15.10	131.27 ± 12.56	135.67 ± 13.85	<0.004*
Diastolic BP (mmHg)		82.24 ± 10.31	80.48 ± 10.02	82.51 ± 9.79	84.57 ± 10.64	0.004*
Patient blood group	A^+ve^	99	51	19	29	0.35**
	B^+ve^	135	54	30	51	
	O^+ve^	91	48	16	27	
	AB^+ve^	45	19	13	13	
Induction regimen	None/Basiliximab/ATG	155/148/67	74/81/17	35/22/21	46/45/29	0.001**
MMF+ Steroid+	Tacrolimus/Cyclosporin	355/15	166/6	72/6	117/3	0.17**

* ANOVA test, ** Chie square test; ABOc, ABO compatible; ABOi, ABO incompatible; BMI, Body mass index; TLC, Total leucocyte count; BUN, Blood urea nitrogen; eGFR, estimated glomerular filtration rate; BP, Blood group; ATG, Anti-thymocyte globulin; MMF, Mycophenolate mofetil.

### Anti-SARS-CoV-2 Spike Protein-Specific IgG Seroconversion Rate Among RTRs

**Figure 1:**
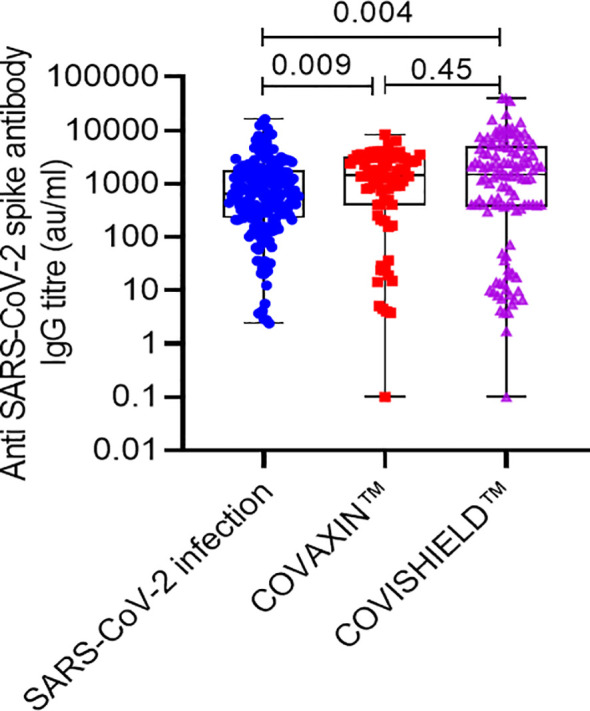
Anti-SARS-CoV-2 spike protein antibody titer in RTRs infected with SARS-CoV-2 or vaccinated with 2 doses of either COVISHIELD™ or COVAXIN™ vaccine.

The overall cumulative seroconversion rate, either due to vaccination or infection, was 85.13% (315/370). The vaccine-associated seroconversion was 80.30% (159/198). The SARS-CoV-2 infection-associated seroconversion was 90.69% (156/172); COVISHIELD™ vaccination-associated seroconversion was 79.2% (95/120), and the COVAXIN™ associated seroconversion was 82.05% (64/78). However, the antibody titer was higher after vaccination than the titer developed only with natural SARS-CoV-2 infection. The median IgG titer in the SARS-CoV-2 infection group was 646.50 AU/ml (IQR: 232.52-1717.42). In the COVAXIN™ group, was 1449.75 AU/ml (IQR: 400.00-3068.55) and in the COVISHIELD™ vaccination group was 1500.51 AU/ml (IQR: 379.47-4938.50). (**Table 2**, **Figure 1**). Further, we compared the percentage of seroconversion and antibodies titer at two different centers. Vaccination associated seroconversion rate was similar between both the center. COVAXIN™ associated seroconversion was 83.9% (47/56) at SGPGIMS compared to 82.05% (64/78) at Medanta Medicity. COVISHIELD™ associated seroconversion in SGPGIMS was 73.8% (31/42) compared to 77% (17/22) in the Medanta Medicity. **Table 3**.

**Table 2:** **^$^** Anti-SARS-CoV-2 antibody titer and seroconversion among renal transplant recipients.

S.No.	SARS-CoV-2 infection(n=172)		COVAXIN™ (n=78)		COVISHIELD™(n=120)		P value
Median titer (Interquartile range)	646.50 (232.52-1717.42)		1449.75 (400.0-3068.55)		1500.51 (379.47-4938.50)		a vs b, p=0.009*; a vs c, p=0.004*; b vs c, p=0.45*
Overall anti-SARS-CoV-2 spike protein antibody seroconversion	Yes 156(90.69%)	No 16(9.3%)	Yes 64(82.05%)	No 14 (17.94%)	Yes 95(79.2%)	No 25(20.83%)	0.017**

$- Indicates seroconversion in patient who had anti SARS-CoV-2 Spike protein antibody titer >50AU/ml. *Kruskal Wallis Test; **Chi Square test.

**Table 3:** **^$^** Seroconversion and antibody titer after vaccination at both center.

Seroconversion and antibody titer in SGPGI Lucknow							
S.No.	SARS-CoV-2 infection(n=172)		COVAXIN^™b^(n=78)		COVISHIELD^™c^(n=120)		P value
Median titer (Interquartile range)	646.50 (232.52-1717.42)		1586.0 (501.95-3000.0)		1572.95 (22.82-7522.75)		a vs b; p=0.033* a vs c; p=0.014* b vs c; p=0.33*
Over all anti-SARS-CoV-2 spike protein antibody seroconversion	Yes 156(90.69%)	No 16(9.3%)	Yes 47(83.9%)	No 9 (16.07%)	Yes 31(73.8%)	No 11(26.19%)	0.012**
Seroconversion and antibody titer in Medanta, Gurugram, India							
Median titer (Interquartile range)			1310.0 (309.05-4000.0)		1400.0 (398.0-4414.52)		0.85*
Over all anti-SARS-CoV-2 spike protein antibody seroconversion			64(82.05%)	14(18%)	17 (77%)	5 (22.7%)	0.76**

$- Indicates seroconversion in patient who had anti SARS-CoV-2 Spike protein antibody titer >50AU/ml. *Kruskal Wallis Test, **Chi Square test.

### Clinical Variables Associated With Seroconversion

The clinical variables associated with seroconversion rate are shown in **Table 4**. Patients with age <45 years had a seroconversion rate of 88.01% (213/242). In the age group 45.1-60 years, seroconversion was 83.18% (94/113), and in patients with age >60 years, seroconversion was only 58.3% (7/12). Older patients had a poor seroconversion rate. There was no impact of BMI, post-transplant interval, gender, blood groups, immunosuppressive regimen, and serum creatinine values on seroconversion (**Table 4**).

**Table 4:** Clinical variables associated with seroconversion among the group.

Characteristics	Variable stratification	Seroconversion		P value
		Yes	No	
Age (years)	<45	213 (88.01%)	29 (11.98%)	0.012
	45.1-60	94 (83.18%)	19 (16.81%)
	>60	7 (58.3%)	5 (41.66%)
	<45	213 (88.01%)	29 (11.98%)
Gender	M	271 (85.5%)	46 (14.51%)	0.67
	F	44 (83.01%)	9 (16.98%)
Post-transplant interval (month)	2-60	133 (86.36%)	21 (13.63%)	0.29
	60.1-120	120 (85.10%)	21 (14.89%)
	120.1-180	38 (79.16%)	10 (20.83%)
	>180	23 (95.8%)	1 (4.16%)
BMI (kg/m^2^)	<18.4	43 (84.3%)	8 (15.68%)	0.38
	18.5-24.99	152 (82.60%)	32 (17.4%)
	25.0-24.99	92 (90.2%)	10 (9.8%)
	>30	28 (84.8%)	5 (15.15%)
Blood group	A^+ve^	86 (86.8%)	13 (13.13%)	0.43
	B^+ve^	110 (81.5%)	25 (18.51%)
	AB^+ve^	38 (84.4%)	7 (15.5%)
	O^+ve^	81 (89.0%)	10 (10.98%)
Blood group compatibility	ABOc	298 (85.8%)	49 (14.12%)	0.13
	ABOi	17 (73.91%)	6 (26.08%)
Serum creatinine (mg/dl)	<1.4	210 (84.33%)	39 (15.6%)	0.64
	>1.4	105 (86.77%)	16 (13.22%)
Immunosuppression	Tacrolimus	302 (85.07%)	53 (14.92%)	1.00
	Cyclosporin	13 (86.6%)	2 (13.3%)

BMI, Body mass index; ABOc, ABO compatible; ABOi, ABO-incompatible.

### Predictor Clinical Variables for Seroconversion on Multivariate Analysis

On multivariate analysis, we observed that the age of the recipients was the significant predictor for seroconversion (B=0.041, Exp (B)=1.04; P=0.004). Other variables like BUN, serum creatinine, total leukocyte count, hemoglobin, BMI, post-transplant gap, eGFR, and trough tacrolimus level were not the predictors of seroconversion (**Table 5**).

**Table 5:** Multivariate analysis predicting seroconversion in RTRs.

Variables	B	Exp (B)	95% CI for EXP (B) (Lower-Upper)	P value
Age (Years)	0.041	1.04	1.03-1.07	0.004
Post-transplant interval (months)	-0.001	0.99	0.99-1.005	0.73
BMI (kg/m^2^)	-0.030	0.97	0.90-1.03	0.36
BUN (mg/dl)	0.005	1.005	0.98-1.03	0.70
Hemoglobin(g/dl)	-0.101	0.90	0.77-1.05	0.197
Serum creatinine(mg/dl)	-0.193	0.82	0.39-1.70	0.60
TLC (X10^3/^µl)	0.008	1.008	0.88-1.14	0.90
eGFR(ml/min)	0.005	1.005	0.980-1.03	0.70
Tacrolimus level(µg/l)	0.13	1.13	0.98-1.31	0.073

TLC, Total leucocyte count; BUN, blood urea nitrogen; eGFR, estimated glomerular filtration rate; BMI, body mass index.

### Side Effects of Vaccination

The major side effects reported by the RTRs for both of the vaccines (COVISHIELD™ and COVAXIN™) were similar. RTRs vaccinated with COVISHIELD™, the mild degree fever was in 13.3%, myalgias in 24.16%, headache in 15.8%, back pain in 4.1%, body- ache in 10%, and giddiness in 12.5%. Whereas RTRs vaccinated with COVAXIN™, the mild degree fever was observed in 10.2%, myalgias in 23.07%, headache in 10.2%, back pain in 3.8%, body ache, and giddiness in 14.10%. In our cohort, no RTRs experienced any major side effects, such as blood clots or thrombotic microangiopathy, similar to the healthy population reported in other studies (18, 19) (**Table 6**).

**Table 6:** Side effects associated with vaccination.

Characteristics	COVISHIELD™(n=120)	COVAXIN™ (n=78)	P value
Myalgia (%)	29 (24.16)	18 (23.07)	0.86
Headache (%)	19 (15.83)	8 (10.2)	0.26
Backpain (%)	5 (4.1)	3 (3.8)	0.91
Body ache (%)	12 (10)	11 (14.10)	0.38
Giddiness (%)	15 (12.5)	11 (14.10)	0.74
Fever (%)	16 (13.3)	8 (10.2)	0.51
Myalgia+ Body ache+ Fever (%)	24 (20)	19 (24.3)	0.47

## Discussion

The current two-center study found that both COVISHIELD™ and COVAXIN™ yielded robust seroconversion up to 80.3% in RTRs at both centers. We also observed that the elderly of more than 60 years had a poor seroconversion rate. The seroconversion rate is inferior to the general population but higher than that reported from mRNA-based vaccines in RTRs from western population studies.

The poor seroconversion is expected in RTRs because of an immunocompromised state because of immunosuppressions like calcineurin inhibitors, mycophenolate mofetil, and corticosteroids. Studies have shown that the use of mycophenolate mofetil significantly hampers the seroconversion after vaccination in RTRs (5, 20). All our patients were on mycophenolate mofetil at the time of sampling. Therefore, antibody formation is less than that expected in the general population (1). However, the SARS-CoV-2 infection alone induced seroconversion in 90% of patients, similar to seroconversion in liver transplant recipients (21). The data from mRNA-based vaccines BNT162b2 (Pfizer–BioNTech, USA) showed a 48% seroconversion rate in RTRs after vaccination, which increased to 49-64% after 3^rd^ dose of vaccination (22, 23). The seroconversion in Indian RTRs appeared much higher than in the European and the USA renal transplant cohorts in both scenarios after infection and vaccination (24–26). The reason for blunted seroconversion in the western population is not known. One of the factors could be the older age of allograft recipients and mainly deceased donor transplantation. Our results found that the seroconversion rate decreased significantly with the increase in the recipient’s age (**Table 2**). Further, multivariate analysis showed age as the best predictor of overall seroconversion. Similar findings were also observed in other studies (24, 25). In our cohort, the median age of the patient was 40.50 years, which was younger than German and UK transplant cohorts (median age 54 and 57 years) (24, 25). The relatively depressed immune system of older people with top-up immunosuppression may have resulted in poor immune response (27). Another important factor associated with seroconversion is the body mass index (BMI) of patients. A poor seroconversion has been reported in obese persons (28). Although, we did not find any difference in seroconversion between lean and obese RTRs. Although, obese patients tended to increase seroconversion rates, similar to the finding by Maria et al. (7). The deceased donor-associated transplant may be another important factor that may influence seroconversion. All patients underwent live-related renal transplantation in our study, and overall, 85.13% (315/370) of patients developed antibodies. In the UK and the German-based cohorts, most patients were undergone deceased donor-organ transplantation (24, 25). Deceased donor-associated allograft recipients usually receive a higher degree of immunosuppression to avoid the risk of rejection. It may be another reason for the lower seroconversion rate in western organ transplant recipients. In our study, the graft dysfunction measured in terms of BUN, Creatinine, and e GFR was not associated with seroconversion. A few studies have shown an association of seroconversion with graft dysfunction. Patients with lower eGFR had poor seroconversion (29, 30). The finding may be because of the fact that the RTRs included in our study had relatively better graft function with a mean serum creatinine value of 1.34 ± 0.60 mg/dl. The longer duration of transplantation indicates a larger duration of immunosuppression, which reduces the chance of antibody formation (29).

One of the exciting findings in our study was the higher seroconversion rate (79.2%) after COVISHIELD™ vaccination at both centers, while the seroconversion rate was only 44% the UK study (31). Besides the age factors, BMI of patients, living versus deceased donor RTS, the seroconversion was also determined by several other factors like genetic makeup of an individual, exposure to antigens, gut microbiota, etc. The vigorous immune response against vaccination in Indian patients may be due to a higher immune response to the pathogens. One of the possible reasons could be Bacille Calmette-Guerin (BCG) vaccination in all RTRs. BCG to prevent tuberculosis disease (TB) is universally given to every child soon after birth in India and other Asian countries with a high prevalence of tuberculosis (32). BCG provokes a non-specific immunity. The cross-protective effects of the BCG vaccine on non-tuberculosis-related diseases are well established (33). The cross-protective effect may be in response to trained innate immune memory (33). It is characterized by non-permanent epigenetic reprogramming of macrophages that leads to increased inflammatory cytokine production and consequently potent immune responses. BCG vaccination was associated with a lower incidence of sepsis and respiratory tract infections that reduced child mortality (34). It has also been observed that BCG vaccination protects against various viral infections such as influenza virus, yellow fever virus, herpes simplex viruses, respiratory syncytial virus, and human papilloma virus (35). The findings confirm a non-targeted beneficial effect of BCG vaccination (33).

The immune system imposes a vigorous response against the SARS-CoV-2 for its clearance, and as a result, there is profuse neutralizing anti-spike IgG antibody formation (36). The higher titer of neutralizing antibody titer in patients with either brand of vaccines in India compared to titers after natural SARS-CoV-2 infection suggests antigenicity of the adjuvants and viral components in sensitization of the immune system for seroconversion is important. This finding gives the clue about designing population-specific vaccines with natural viral components and potent antigenic adjuvants. The seroconversion rate was higher with natural infection but the antibody titer was lower than that occurred after vaccination. The lower antibody titer may be due to the waning of the antibody over time (3, 37). It is speculated that whole virus-based vaccines may induce the robust seroconversion and elicitation of antibody titer as reflected in our finding (**Table 2**) (10).

As SARS-CoV-2 virus induce innate immune components leading to proinflammatory cytokines secretion such as IL-1β, and IL-18 (Cytokine storm) (38). A higher inflammatory state leads higher seroconversion rate after vaccination in solid organ transplant recipients (39). A study showed a 100% seroconversion rate in RTRs after the first dose of mRNA-based vaccination in previous SARS-CoV-2 infected patients (13). Again, suggesting the importance of the co-stimulating effect of viral components in seroconversion. Alternatively, reducing the immunosuppressive dose may help in raising the antibody titer. Although, it may increase the chances of rejection. Alternatively, using more antigenic material in vaccine preparation may improve the vaccine efficacy for these patients. The present two-center study confirms the finding of our single-center study of higher seroconversion rate with COVISHIELD™ and COVAXIN™ in living donor renal transplant patients in India (10). The side-effects profile suggests the safety of these vaccines in RTRs. It is also prudent to understand whether a single dose of vaccination is sufficient in a previously infected person or whether complete vaccination with two doses is required. It is particularly important for low-middle income and resource-limited countries where vaccination of the entire population is still a dream. Owing to the fourth wave of SARS-CoV-2 infection, vaccination with either of these two vaccines will be helpful in elicitating the effective SARS-CoV-2 neutralizing antibody in population.

## Conclusions

Both infection and vaccination induce robust antibody formation in RTRs. SARS-CoV-2 infection induces a higher seroconversion but poor antibody titer. The vaccines induce more elevated antibodies titer than natural infection. The response rate in Indian RTRs appears better than the western population.

## Data Availability Statement

The raw data supporting the conclusions of this article will be made available by the authors, without undue reservation.

## Ethics Statement

The studies involving human participants were reviewed and approved by Institute Ethics Committe, SGPGI. The patients/participants provided their written informed consent to participate in this study.

## Author Contributions

NP, BY and SB Conceived the project, counselled patients for sample donation, supervised the progress of project, edited the final draft of manuscript. BY collected the sample, analyzed the IgG titer, analyzed the data, wrote the initial draft of manuscript NM collected the samples and helped in IgG titer analysis. DY, SG, and AK helped in sample collection and analysis of the IgG titer. RK, DB, MY, MB, and AK coordinated with the patients for sample donation and reviewed the final draft of manuscript. All authors contributed to the article and approved the submitted version.

## Conflict of Interest

The authors declare that the research was conducted in the absence of any commercial or financial relationships that could be construed as a potential conflict of interest.

## Publisher’s Note

All claims expressed in this article are solely those of the authors and do not necessarily represent those of their affiliated organizations, or those of the publisher, the editors and the reviewers. Any product that may be evaluated in this article, or claim that may be made by its manufacturer, is not guaranteed or endorsed by the publisher.
